# Large Cell Neuro-Endocrine Carcinoma of the Lung: Current Treatment Options and Potential Future Opportunities

**DOI:** 10.3389/fonc.2021.650293

**Published:** 2021-04-15

**Authors:** Miriam Grazia Ferrara, Alessio Stefani, Michele Simbolo, Sara Pilotto, Maurizio Martini, Filippo Lococo, Emanuele Vita, Marco Chiappetta, Alessandra Cancellieri, Ettore D’Argento, Rocco Trisolini, Guido Rindi, Aldo Scarpa, Stefano Margaritora, Michele Milella, Giampaolo Tortora, Emilio Bria

**Affiliations:** ^1^ Comprehensive Cancer Center, Fondazione Policlinico Universitario Agostino Gemelli IRCCS, Roma, Italy; ^2^ Medical Oncology, Università Cattolica del Sacro Cuore, Roma, Italy; ^3^ Department of Diagnostics and Public Health, Section of Anatomic Pathology, University of Verona, Verona, Italy; ^4^ Section of Oncology, Department of Medicine, University of Verona Hospital Trust, Verona, Italy; ^5^ Institute of Pathology, Università Cattolica Del Sacro Cuore, Fondazione Policlinico Universitario Agostino Gemelli IRCCS, Roma, Italy; ^6^ Thoracic Surgery, Fondazione Policlinico Universitario Agostino Gemelli IRCCS, Università Cattolica del Sacro Cuore, Rome, Italy; ^7^ Interventional Pulmonology Unit, Fondazione Policlinico Universitario Agostino Gemelli IRCCS, Università Cattolica del Sacro Cuore, Rome, Italy

**Keywords:** large cell, lung cancer, review, neuro-endocrine, treatment

## Abstract

Large-cell neuroendocrine carcinomas of the lung (LCNECs) are rare tumors representing 1–3% of all primary lung cancers. Patients with LCNEC are predominantly male, older, and heavy smokers. Histologically, these tumors are characterized by large cells with abundant cytoplasm, high mitotic rate, and neuroendocrine immunohistochemistry-detected markers (chromogranin-A, synaptophysin, and CD56). In 2015 the World Health Organization classified LCNEC as a distinct subtype of pulmonary large-cell carcinoma and, therefore, as a subtype of non-small cell lung carcinoma (NSCLC). Because of the small-sized tissue samples and the likeness to other neuroendocrine tumors, the histological diagnosis of LCNEC remains difficult. Clinically, the prognosis of metastatic LCNECs is poor, with high rates of recurrence after surgery alone and overall survival of approximately 35% at 5 years, even for patients with early stage disease that is dramatically shorter compared with other NSCLC subtypes. First-line treatment options have been largely discussed but with limited data based on phase II studies with small sample sizes, and there are no second-line well defined treatments. To date, no standard treatment regimen has been developed, and how to treat LCNEC is still on debate. In the immunotherapy and targeted therapy era, in which NSCLC treatment strategies have been radically reshaped, a few data are available regarding these opportunities in LCNEC. Due to lack of knowledge in this field, many efforts have been done for a deeper understanding of the biological and molecular characteristics of LCNEC. Next generation sequencing analyses have identified subtypes of LCNEC that may be relevant for prognosis and response to therapy, but further studies are needed to better define the clinical impact of these results. Moreover, scarce data exist about PD-L1 expression in LCNEC and its predictive value in this histotype with regard to immunotherapy efficacy. In the literature some cases are reported concerning LCNEC metastatic patients carrying driver mutations, especially EGFR alterations, showing targeted therapy efficacy in this setting of disease. Due to the rarity and the challenging understanding of LCNEC, in this review we aim to summarize the management options currently available for treatment of LCNEC.

## Introduction

Large cell neuroendocrine carcinoma of the lung (L-LCNEC) represents a rare but highly aggressive NSCLC with neuroendocrine differentiation, accounting for 2–3% of all lung cancers. Patients are often male and older with heavy smoking history. In 2015, the World Health Organization classified LCNEC as a distinct subtype of pulmonary large-cell carcinoma and, therefore, as a neuroendocrine non-small cell lung carcinoma (NSCLC) (1–4). Histologically, LCNEC has a neuroendocrine morphology including organoid or trabecular patterns, rosette-like structures, or peripheral palisading. Tumor cells exhibit abundant (often eosinophilic) cytoplasm, prominent nucleoli, large areas of necrosis, and high mitotic rate. Radiologically, LCNEC can be solitary or multiple and is often localized in the lung periphery (5–7). Clinically, LCNEC frequently spreads metastases to the liver, brain and/or bone. The prognosis of patients with LCNEC is poor, and stage by stage, survival curves of L-LCNEC and SCLC almost overlap, with high rates of recurrence even for patients with early stage disease and overall survival dramatically shorter compared with other NSCLC subtypes (8, 9). Regarding all stages, 5-year survival rate and 5-year disease-free survival rate are about 35 and 27% respectively; great part of relapses occurred within the first 2-year follow-up (10, 11). Treatment strategies for this tumor are largely discussed, and to date, no standard management exists, especially for advanced stages due to its rarity resulting in a scarce accrual in clinical trials. Resectable LCNEC is treated by surgical excision; for unresectable LCNEC with locally advanced or metastatic disease optimal systemic treatment has not been established, and it is largely discussed since patients may be treated with SCLC-regimen (etoposide/platinum) or NSCLC regimen according to the American Society of Clinical Oncology (ASCO) guidelines (12). Nevertheless, LCNEC appears overall more aggressive than most NSCLC and less responsive to SCLC-regimens (13, 14). With regard to the tricky treatment choice, recent data suggest that RB1 expression could represent a potential biomarker to select the best treatment for LCNEC patients (RB1 loss should guide toward SCLC-chemotherapy; on the other hand, LCNEC showing intact RB1 expression should be treated with NSCLC-regimens) (15, 16). No standard second-line treatment exists, and a few data are available regarding patients treated with checkpoint inhibitors after first-line progression, with a moderate efficacy reported (17).

Based on the difficult classification of LCNEC, several efforts have been done for a deeper understanding of its molecular characteristics. Thereby some studies have detected some subtypes of LCNEC are more sensitive to chemotherapy, the existence of potential targetable gene mutations and the presence of transcriptomic subtypes with specific genomic alterations that correlate with prognosis (15, 18).

In the last decade, targeted therapy for ‘oncogene addicted’ disease and immunotherapy for PD-L1 positive patients have deeply reshaped treatment strategies in NSCLC. Instead, for rare tumors as LCNECs, clinical trials are difficult to conduct, and to date, there is not a clear indication for immunotherapy or targeted therapy in LCNEC patients. Only a few studies in literature report patients treated with immunotherapy, mainly pretreated patients, showing a higher percentage of PD-L1 expression in comparison to other neuroendocrine tumors (especially SCLC) and a moderate efficacy of checkpoint inhibitors (19, 20). Regarding ‘oncogene addiction’ in LCNEC, some cases are reported in ‘pure’ LCNEC (without adenocarcinoma component) metastatic patients carrying driver mutations, including EGFR alterations (21, 22), ALK rearrangements (23), and KRAS mutations (24, 25). Although infrequent, the driver mutations reported in LCNEC draw a sharp contradistinction with classic SCLC, which in the ‘pure’ form is consistently devoid of adenocarcinoma-type driver mutations (26, 27).

Considering the above discussion, there is an emerging need for an agreement on the best management to adopt for this aggressive lung cancer histotype. The aim of this review is to point out the current clinical and molecular findings and to highlight the potential treatment strategies for LCNEC patients.

## Histo-Pathological Features

Histologically, LCNEC diagnosis may be challenging. Any amount of morphologically recognizable adenocarcinoma, squamous cell carcinoma, giant cell carcinoma, or spindle cell carcinoma in combination with LCNEC is sufficient for the diagnosis of combined LCNEC with the corresponding component (28). Neuroendocrine markers (synaptophysin, chromogranin A or CD56) are typically present and are diriment for diagnosis, although they are not specific of LCNEC and may be observed in other neuroendocrine tumors. Indeed, the differentiation of LCNEC from small cell lung carcinoma (SCLC) or atypical carcinoid can be challenging, especially in small biopsy sample specimens (29). For an accurate diagnosis, LCNEC requires the presence of neuroendocrine features identified using light microscopy, neuroendocrine differentiation confirmed by electron microscopy and/or immunohistochemistry (synaptophysin, chromogranin A or CD56 positivity), mitoses ≥11 per 2 mm^2^, and non-small cell cytomorphology with abundant eosinophilic cytoplasm and prominent nucleoli (30). By immunohistochemistry (IHC), LCNEC is positive for chromogranin and CD56 in greater than 80% of cases, and synaptophysin and TTF-1 in approximately 40 to 50% of cases (31). Because of the difficult diagnosis in biopsies or cytology specimens, LCNEC may be suspected when the tumor shows non-small cells, high-grade neuroendocrine features with no overt squamous or glandular differentiation and expresses more than one neuroendocrine marker (synaptophysin, chromogranin, and/or CD56) (16). Considering the above discussion, distinguishing LCNEC from SCLC represents the most insidious challenge due to the similar neuroendocrine morphology and biomarkers’ expression. Because complete loss of RB1 expression is found in greater than 95% of SCLCs and approximately 50% of LCNECs, intact RB1 expression in equivocal cases may favor LCNECs over SCLCs (25, 32). Recently, a new category of thoracic tumors has been described, designated as SMARCA4-deficient undifferentiated thoracic tumor (SD-UTT) (33–35). Histologically, these tumors appear undifferentiated and show high-grade cell with rhabdoid morphology and synaptophysin immunoreactivity in 70% of cases (36). SD-UTTs do not present neuroendocrine features but are frequently characterized by high mitotic rate and extensive necrosis. Thus, in a crushed biopsy, these tumors may mimic LCNEC laying a pitfall for a correct diagnosis (28).

## Molecular Characteristics of LCNEC

Despite the remarkable advances in understanding the molecular landscape of lung adenocarcinoma, to date LCNEC has remained poorly characterized due to its low prevalence. Nevertheless, considering its aggressive features, a series of studies have been conducted to investigate the molecular characteristics of LCNEC. Rekhtman et al. (25), through next-generation sequencing, have shown that these tumors are molecularly heterogeneous and can be classified into two major subsets: small cell-like LCNEC (SCLC-like LCNEC) and non-small cell-like LCNEC (NSCLC-like LCNEC). SCLC-like LCNEC subset was characterized primarily by RB1 and TP53 inactivation, whereas NSCLC-like LCNEC subset was associated with KRAS, serine/threonine kinase 11 gene (STK11)/kelch-like ECH associated protein 1 gene (KEAP1) mutations alone or concurrently with TP53 mutations. Additional less common molecular alterations seen almost exclusively in the SCLC-like LCNEC included PTEN mutations and MYCL1 amplification, and those seen exclusively in the NSCLC-like LCNEC involved MAP2K1, ERBB2, BRAF, and CDKN2A genes. Furthermore, a small subset of carcinoid-like LCNECs was identified, which was characterized by MEN1 alterations and lack of RB1/TP53 alterations. With regard to morphologic features, as expected, SCLC-like LCNEC subset tends to have a spectrum of characteristics closer to SCLC than NSCLC-like LCNEC subset, for example higher Ki-67 rates and smaller cell size (25).

A study in 75 LCNECs conducted by George et al. (37) has confirmed the existence of two LCNEC subtypes, one (type II) characterized by the concurrent inactivation of TP53 and RB1 (42%) and one (type I) with TP53 and STK11/KEAP1 alterations (37%) (**Table 1**).

**Table 1:** Differences between LCNEC and SCLC.

Characteristics	LCNEC	SCLC
*Incidence*	2–3%	15–20%
*Clinical characteristics*	Male, elderly, smokers	Male, elderly, smokers
*Site of disease*	Mostly peripheral	Central
*Histological characteristics*	Large cells Abundant cytoplasm Prominent nucleoli Abundant necrosis	Small cells Scarce cytoplasm No prominent nucleoli Abundant necrosis
*IHC markers positivity*	Synaptophysin, Chromogranin A and/or CD56	Synaptophysin, Chromogranin A and/or CD56
*Molecular subtypes*	SCLC-like (RB1 and TP53 inactivation) NSCLC-like (STK11, KEAP1 and/or TP53 mutations)	RB1 and TP53 inactivation
*Early stage treatment*	Surgery and adjuvant chemotherapy	Chemo-radiotherapy
*Advanced stage treatment*	Not established (mainly platinum-etoposide)	Platinum-etoposide plus immunotherapy combination
*Five-year survival*	35%	<15%

LCNEC, large cell neuroendocrine carcinoma; SCLC, small cell neuroendocrine carcinoma; NSCLC, non-small cell lung cancer; IHC, immunohistochemistry.

Particular attention was paid to delta-like ligand 3 (DLL3) that is an inhibitory Notch-ligand highly expressed in SCLC and LCNEC (38, 39). It represents a new potential therapeutic target, and it has recently been reported that DLL3 mRNA expression is particularly upregulated in the LCNEC subgroup with STK11/KEAP1 and TP53 co-mutations, in contrast to lower expression levels in RB1 and TP53 co-mutated LCNEC. Indeed, two gene expression profiles have been found: a DLL3^high^/Notch^low^ profile with high expression levels of neuroendocrine genes (synaptophysin, chromogranin A) in LCNEC with TP53 and STK11/KEAP1 mutations and, on the other hand, a DLL3^low^/Notch^high^ gene expression profile and lower expression levels of neuroendocrine genes in LCNEC with TP53 and RB1 mutations. The high percentage of DLL3 positive SCLC and LCNEC combined with low or non-detectable DLL3 levels in healthy tissue make DLL3 attractive for targeted therapy (40).

Considering the evidence that high-grade neuroendocrine carcinomas may evolve from preexisting carcinoids (41), Simbolo et al. have investigated the transcriptomic relationship between atypical carcinoids (ACs) and LCNECs and have demonstrated that ACs and LCNECs comprise three different molecular diseases of potential clinical relevance: one AC-enriched group (C3) in which MEN1 inactivation plays a major role, one LCNEC-enriched group (C1) whose hallmark is RB1 inactivation, and one group (C2) with intermediate molecular features. These data support a progression of malignancy that may be traced by using combined molecular and immunohistochemical analysis (18). Regarding transcriptional profile, genes involved in the mitotic spindle and MYC targets were enriched in C1, consistent with recurrent MYC copy gain found in this cluster (18).

Another study has highlighted that the differences between carcinoids and high-grade carcinomas (LCNEC and SCLC) reside in the prevalence rates of the most frequently mutated genes (inactivating alterations of TP53 and RB1 were enriched in carcinomas, whereas MEN1 alterations were almost exclusive to carcinoids), with the exception of SMARCA2 which results in alteration in the LCNEC only.

Actually, the same gene alterations have been found in LCNEC and in low-grade tumors with a lower prevalence rate, suggesting the existence of a progression of malignancy from carcinoids to high-grade neuroendocrine carcinomas. Moreover, in this study, PI3K/AKT/mTOR pathway alterations have been identified in LCNEC, in particular PIK3CA mutations (11%), PIK3CA copy number variation (CNV) (33.3%), and RICTOR CNV (37%). At last, survival analysis has shown that mutations of RB1 and copy gain of telomerase reverse transcriptase gene (TERT) are independent predictors of poor prognosis in patients with neuroendocrine tumor (regardless of subtype). These data underlie the relevance of molecular profiling in neuroendocrine tumors and, in particular, in LCNEC patients, to provide better diagnostic classification and prognostic stratification that could be helpful for their clinical management (42).

Derks et al. have evaluated whether the two LCNEC subtypes previously identified (RB1 mutated *versus* RB1 wild-type) have a predictive value on chemotherapy outcome. They have assessed that LCNEC patients carrying a wild-type RB1 gene or expressing the RB1 protein benefit more from platinum-based chemotherapy plus gemcitabine or taxane treatment than from standard SCLC chemotherapy (platinum plus etoposide) (15). This result confirms that molecular alterations may guide the best treatment strategy for these patients. With regard to the therapeutic implications for LCNEC subtypes, a recent study has demonstrated that patients with SCLC-like LCNEC had a shorter OS than those with NSCLC-like LCNEC despite higher response rate (RR) to chemotherapy. Furthermore, treatment with etoposide-platinum was associated with superior response and survival in SCLC-like LCNEC compared to pemetrexed–platinum and gemcitabine/taxane–platinum doublets, while treatment with gemcitabine/taxane–platinum led to a shorter survival compared to etoposide-platinum or pemetrexed–platinum in NSCLC-like LCNEC patients. In summary, this study has stressed the concept that genomic subtyping has a potential role in prognosis prediction and therapeutic decision for patients with LCNEC (43).

Miyoshi et al. have performed targeted capture sequencing of all the coding exons of 244 cancer-related genes on 78 LCNEC samples (including 10 LCNECs combined with NSCLC) and have compared genomic alterations with those of 141 SCLCs. The authors have found a relatively high prevalence of inactivating mutations in TP53 (71%) and RB1 (26%), but the mutation frequency in RB1 was lower than that in SCLCs (40%). Additionally, genetic alterations in the PI3K/AKT/mTOR pathway were detected in 15% of the LCNEC: PIK3CA 3%, PTEN 4%, AKT2 4%, RICTOR 5%, and mTOR 1%. Other activating alterations were detected in KRAS (6%), FGFR1 (5%), KIT (4%), ERBB2 (4%), and EGFR (1%). Although the frequency of each mutation is low, the overall rate is significant, suggesting that molecular profiling is warranted in LCNEC for potential targeted therapies (44).

Pelosi et al. have studied the role of E-cadherin/*β*-catenin system dysregulation in pulmonary neuroendocrine tumors. They have shown that changes in E-cadherin/*β*-catenin expression patterns are common in lung neuroendocrine tumors, with either subcellular redistribution and/or down-regulation and that the subcellular compartmentalization of *β*-catenin is profoundly altered in LCNEC. Moreover, E-cadherin/*β*-catenin system alterations are able to induce the activation of epithelial–mesenchymal transition (EMT) in a subset of LCNEC and SCLC (45–47).

Another field of interest concerns the epigenetic alterations that might be involved in LCNEC development. In particular, Li et al. have demonstrated that the progressive loss of histone H4 acetylation at lysine16 (H4KA16) and trimethylation at lysine 20 (H4KM20) from low to high grade lung neuroendocrine tumors reflects the degree of differentiation and proliferative activity (48). Also methylation patterns have inspired interest because of their correlation with gene expression in lung neuroendocrine cancers (49).

Despite the efforts made to deeply understand LCNEC molecular features, a great part of its complexity has yet to be explored. Several studies performed have allowed the identification LCNEC subtypes with different genomic profiles and potential targetable gene alterations. Some correlations have also been found between LCNEC subtypes and response to chemotherapy regimens, giving interesting suggestions regarding how to select the best treatment for LCNEC patients. Furthermore, the extensive genomic profiling has detected the existence of numerous molecular alterations that, taken together, are not uncommon and lays the foundations for further developing targeted therapy. All these findings underlie the relevance of performing an extensive genomic profile in LCNEC to facilitate patients’ management in terms of both prognostic implications and treatment selection.

## Early Stage: Role Of Surgery and Radiotherapy

Despite its similarities to SCLC, surgery represents the cornerstone of the treatment of localized LCNEC (13, 50, 51). The low prevalence of this subgroup of NSCLC limits the quality of available data; in fact this recommendation mostly derives from retrospective studies and case series. Despite the absence of strong evidence, patients with resectable LCNEC should undergo surgery as a primary treatment (52).

Surgery represents a positive independent prognostic factor for OS, as demonstrated by Cao et al. in a large retrospective study including 1,530 patients with all-stages LCNEC. In their analysis, better outcomes were associated with lobectomy/bilobectomy (HR 0.357, p < 0.001) than with wedge resection/segmentectomy (HR 0.526, p < 0.001) or with pneumonectomy (HR 0.491, p < 0.001) (53). Regarding the surgical extent, in the subgroup analysis of the retrospective study by Wakeam et al. sub-lobar resection for stage I LCNEC was, once again, correlated with worse OS than lobectomy (HR 1.40, p < 0.001) (54). Another large retrospective study from Gu et al. showed that surgery, when feasible, significantly and independently improved OS compared to a cohort of not surgically treated patients (adjusted with propensity score matching—PSM—method); this was demonstrated for stages I–II (p = 0.000), for stage IIIA (p = 0.001), and even for stage IIIB (p = 0.017), although the high recurrence rate after surgery alone justifies the need for a multimodal treatment in all-stage LCNEC (55). Several smaller series are in line with the above-mentioned studies, as demonstrated by Girelli et al. (56), Lowczak et al. (57), and Zacharias et al. (58). The latter study also demonstrated a possible role for systematic mediastinal nodal dissection in improving outcomes in LCNEC, but this finding might be explained by a more accurate staging of the disease.

Eichhorn and colleagues analyzed clinical and immunohistochemical predictors of survival after surgery in a retrospective cohort of 57 patients: a poorer prognosis was associated with advanced stage and advanced nodal involvement; a negative trend was also associated with the expression of neuroendocrine immunohistochemical markers: the expression of CgA, CD56 or both was a predictor for a significantly worse relapse-free survival and, not significantly, for a worse OS (59).

The role of radiotherapy (RT) in LCNEC is still unclear due to conflicting evidence. In the large retrospective study by Raman et al., patients with stage I LCNEC (n = 3,371) were treated with surgery (96%) or with stereotactic body radiation therapy (SBRT) (4%); in a multivariate analysis, OS was better in patients undergoing surgery (5 y OS 50 *versus* 27%, HR 0.7). In stage II and IIIA patients, definitive chemoradiation was associated with worse survival than surgery, although 40% of patients in the surgery cohort with stage IIIA underwent adjuvant chemotherapy and 14% induction chemotherapy (60). SBRT was again compared to surgery in patients with T1–2 N0 LCNEC in the retrospective analysis by Lo and colleagues: after adjusting the cohorts with PSM method, median OS was 34.6 months in the SBRT group and 57.2 months in the surgical group with corresponding 5 y OS of 25 *versus* 48% (P <  0.0001) (61).

Wegner et al. retrospectively compared patients with T1–2 N0 LCNEC not suitable for surgery treated with SBRT or conventional fractionated radiotherapy (CFRT), and the results favored SBRT (median OS of 34.7 *versus* 23.7 months; p = 0.02) (62). Gu and colleagues demonstrated that patients with stage I–III LCNEC not suitable for surgery achieved a better prognosis with the combination of definitive chemo-radiation than with chemotherapy alone (p = 0.003) (55).

In the post-operative setting, two large retrospective trials (54, 63) showed that, for patients with early stage LCNEC, RT did not give additional benefit in OS. Jiang et al. reported a possible detrimental effect of RT I–III LCNEC in resected patients (median OS 27 *versus* 44 months with surgery alone), but a limitation of this study is the lack of information on the possible use of adjuvant chemotherapy (64). These findings, together with the recently presented results of LungART trial in NSCLC with N2 nodal involvement, underlined the need to be cautious in considering post-operative RT (65).

In conclusion, differently from SCLC, surgery represents the first option for resectable LCNEC; RT might be offered to ineligible patients, especially as a part of a multimodal treatment including chemotherapy.

## Early Stage: Role of Adjuvant Chemotherapy

The prognosis of LCNEC remains poor, compared to other histotypes of NSCLC, even after radical surgery, highlighting the importance of a multimodal approach particularly in the earliest stages (60). In a retrospective analysis, Iyoda et al. found that the 5-year OS of patients with completely resected stage I NSCLC was 54.5% for LCNEC and 89.3% for other histotypes (p = 0.0012) (66). Other studies reported 5-year survival rates ranging as follows: stage I 33–62%, stage II 18–75%, stage III 8–45% (14). Tumor recurrences tend to develop early: 64% within 1 year after surgery, 91% within 3 years (10). Adjuvant chemotherapy (AC) has been investigated with the aim to improve recurrence-free survival and OS.

The rarity of this histology of NSCLC makes it difficult to perform a prospective trial on patients with completely resected LCNEC. In fact, up to now, there is only a small single-arm prospective study which included only patients with LCNEC, designed by Iyoda and colleagues; this trial evaluated the efficacy of AC with cisplatin plus etoposide on fifteen patients who underwent lobectomy with lymph node dissection. The control group derived from retrospective data. The results were in favor of the adjuvant arm, with a 5 y OS of 88.9 *versus* 47.4% in the retrospective arm (p = 0.0252) (67). Another prospective trial including forty patients with resected high-grade NEC of the lung (both LCNEC and SCLC) showed a positive trend on survival given by a post-operative chemotherapy with the combination of cisplatin and irinotecan for 4 cycles (68). A recently published phase III trial in the same population of high grade NEC of the lung was designed to show the superiority of cisplatin plus irinotecan over cisplatin plus etoposide as adjuvant regimen; the trial was interrupted at the second interim analysis due to futility; in fact no statistically significant difference was found between the two arms: at a median follow-up of 24.1 months, the 3-year relapse-free survival was 65.4% for etoposide plus cisplatin and 69% for irinotecan plus cisplatin (HR 1.076, p= 0.619) (69).

Wakeam and colleagues, in a retrospective study with a cohort of 1,770 patients with LCNEC, showed that AC was associated with better OS especially if tumor dimension was greater than 3 cm (5 y OS 59.8 *versus* 42.1%) and if chemotherapy was administered within 3–6 months after surgery; on the contrary, no advantages were seen if tumor was smaller than 2 cm and if chemotherapy was started after 6 months from surgery (54). In the retrospective analysis by Raman et al. 2,642 patients with stage I LCNEC were included in order to investigate AC in early stages: although a significant increase in OS was observed in the overall population (p = 0.002), the subgroup analysis showed no survival benefit for patients with stage IA (63). Conversely, the retrospective study by Kujtan and colleagues conducted on 1,232 patients with stage I LCNEC showed that AC conferred a significant benefit in OS both in stage IA (HR 0.64, p = 0.006) and in stage IB (HR 0.43, p < 0.001) (70).

A positive trend of AC in stage I LCNEC was confirmed by Veronesi et al. (71); Rossi et al. also demonstrated how SCLC-type adjuvant chemotherapy (platinum plus etoposide) was associated with better outcomes than NSCLC-type regimens (platinum plus taxane/vinorelbine/gemcitabine), with a median OS (mOS) of 42 *versus* 11 months (p < 0.001) (72).

Gu and colleagues analyzed a cohort of 2,594 patients with all stages LCNEC who underwent different treatment modalities; not only the benefit of adjuvant chemotherapy was observed from stage I to stage III, but also the combination of surgery and chemotherapy represented the best strategy for these patients compared to surgery plus radiotherapy or surgery plus chemoradiation (55). Sarkaria et al. confirmed a positive effect of AC and possibly, of neo-adjuvant chemotherapy, in patients from stages IB to IIIA (73).

In conclusion, although these studies showed a positive impact on survival by AC, no general recommendation can be made due to the retrospective nature of most of the data and, most importantly, due to the lack of information on the regimens used (**Table 2**).

**Table 2:** Available studies evaluating adjuvant chemotherapy in LCNEC.

Authors	Study	Patients	Treatment	Outcomes
*Iyoda et al.*	Prospective (single arm)	50	Cisplatin-Etoposide	5 y OS: 88.9%
*Kenmotsu et al.*	Prospective (single arm)	40 (23 LCNEC)	Cisplatin-Irinotecan ×4 cycles	3 y OS: 81% (86% LCNEC)
*Kenmotsu et al.*	Prospective (two arms)	221	Cisplatin-Irinotecan *vs* Cisplatin-Etoposide	3 y RFS: 69 *vs* 65.4% (p = 0.619)
*Wakeam et al.*	Retrospective	1770	S + AC (463) *vs* S	5 y OS: 59.2 *vs* 45.3%
*Raman et al.*	Retrospective	2642 (stage I)	S + AC (481) *vs* S (2,161)	mOS: 81 *vs* 65 m (p = 0.002) Stage IA HR 0.92 Stage IB HR 0.67
*Kujtan et al.*	Retrospective	1232 (stage I)	S + AC (275) *vs* S (957)	5y OS: 64.5% vs 48.4% (p < 0.001) Stage IA HR 0.63(*) Stage IB HR 0.55(*)
*Veronesi et al.*	Retrospective	144	NAC or AC	5 y OS: 42.5%
*Rossi et al.*	Retrospective	83	SCLC-based AC vs NSCLC-based AC	mOS 42 *vs* 11 m (p = 0.0001)
*Gu et al.*	Retrospective	2,594 (569 stage I)	S *vs* S + AC *vs* S + RT *vs* S + CRT	S + AC best option (p = 0.03)
*Sarkaria et al*	Retrospetive	100	NAC or AC (platinum-based)	mOS 7.4 *vs* 2 years

S, Surgery; AC, adjuvant chemotherapy; NAC, neoadjuvant chemotherapy; RFS, relapse free survival; mOS, median overall survival.

*propensity-matched.

## Locally-Advanced Disease

Patients with unresectable stage III LCNEC should receive, in accordance with the latest guidelines, a multimodal treatment comprising systemic chemotherapy and radiotherapy (52). As already mentioned for the early stage, most recommendations derive from retrospective data and from the translation of results of studies conducted on SCLC and NSCLC patients.

Limmonik et al. have recently published the results of a large retrospective trial of 5,797 patients with locally advanced LCNEC in which they compared the use of definitive chemo-radiotherapy (CRT) *versus* chemotherapy alone. Despite the limitations of the study due to the retrospective nature and the lack of information about the regimen of chemotherapy administrated, the results were in favor of CRT with a mOS of 16.1 *versus* 11.9 months in the chemotherapy group (p < 0.0001) (74).

These results were confirmed in the retrospective study conducted by Gu and colleagues: the subgroup of LCNEC patients not suitable for surgery had better outcomes with the combination of definitive CRT than with chemotherapy alone (p = 0.003); nevertheless, this analysis included not only locally advanced LCNEC but also inoperable stage I and II patients (55).

Shimada et al. retrospectively evaluated a cohort of 25 patients with unresectable high-grade neuroendocrine carcinomas of the lung: those who underwent CRT had higher objective response rate (ORR) than those treated with chemotherapy alone (86 *versus* 61%), but the sample size was too small to draw any conclusion (75).

With regard to the regimen choice, no prospective data are available. Large clinical trials conducted for stage III NSCLC patients rarely included a very low proportion of LCNEC. In addition, the activity and efficacy of SCLC-like and NSCLC-like regimens have been better investigated in the metastatic setting, as discussed below. However, two small retrospective studies involving both locally advanced and metastatic LCNEC showed that platinum-based SCLC-like regimens could reach response rates similar to those of SCLC (75, 76).

In conclusion, considering the few available data, concurrent chemoradiation (CRT) with four cycles of platinum and etoposide could represent a potential appropriate treatment for stage III unresectable LCNEC (77).

## Advanced Disease: Systemic Chemotherapy

Historically, data on systemic therapeutic approaches to stage IV LCNEC has been conflicting due to the likeness of this histotype to SCLC neuroendocrine features and, at the same time, the classification as NSCLC. The gold standard chemotherapy for advanced or metastatic LCNEC is still debated (75, 76, 78–82), and due to the rarity of LCNEC subtype, there are no clinical trials tailored for LCNEC patients. Platinum compounds and taxanes have established activity in advanced disease (76, 78), but the prognosis remains poor with a median overall survival (OS) of 8–12 months (79, 82). Thus, there is an imperative need for prospective studies of novel compounds. Nevertheless, considering LCNEC’s biological relation to SCLC, four to six cycles of etoposide combined with cisplatin or carboplatin chemotherapy are generally recommended for the advanced stage (2, 10). Sun et al. retrospectively evaluated whether advanced LCNEC should be treated similarly to SCLC or NSCLC. Of 45 patients, 11 were treated with SCLC-regimens and 34 with NSCLC-standard chemotherapy. The median PFS was 6.1 *versus* 4.9 months (p = 0.41), the median OS was 16.5 *versus* 9.2 months (p = 0.10), and the ORR was 73 *versus* 50% (p = 0.19) in the SCLC and NSCLC regimen groups, respectively. Even for second-line treatment the most common drugs used in SCLC (taxanes or irinotecan) showed a clear superiority to those used in NSCLC (pemetrexed, gefitinib or erlotinib) (78). Le Treut et al. conducted a phase II prospective study on 42 advanced LCNEC patients treated with cisplatin and etoposide doublets. The median PFS and median OS were 5.2 and 7.7 months, respectively (79). Igawa et al. evaluated the clinical efficacy of chemotherapy for unresectable LCNEC, showing that outcomes are comparable to that for SCLC extensive disease (80). Similarly, in the study of Yamazaki et al. twenty LCNEC patients were enrolled, of which nine received cisplatin and etoposide; six cisplatin, vindesine, and mitomycin; four cisplatin and vindesine; and one cisplatin alone. Patients treated with platinum doublets achieved an ORR comparable to that in SCLC (76). Shimada et al. retrospectively evaluated 25 LCNEC patients treated with chemotherapy or chemo-radiotherapy as first-line treatment and compared their data with those of 180 SCLC patients. The ORR was 86 and 98% in LCNEC and SCLC respectively; the 1-year survival rate of LCNEC was 34 *versus* 49% in SCLC patients (p = 0.84). With regard to the efficacy of second-line chemotherapy, it appeared significantly lower for LCNEC (ORR of 17% in LCNEC patients *versus* 45% in SCLC patients) (75). In their study, Fujiwara et al. demonstrated some activity of irinotecan and paclitaxel with or without platinum in patients with LCNEC (median OS 10.3 months and 1-year survival rate 47.6%) (81). Niho et al. conducted a phase II study to evaluate irinotecan plus cisplatin combination in patients with advanced LCNEC; 30 patients with LCNECs and 10 with SCLCs were enrolled. The ORR and median OS was 40 *versus* 80% (p = 0.0823) and 12.6 *versus* 17.3 months (p = 0.047) for LCNEC group and SCLC group, respectively. The authors conclude that this regimen was active in LCNEC, but ORR and OS were inferior to those of SCLC, showing a minor chemo-responsiveness of LCNEC (82). Rossi et al. (72) analyzed 83 cases of LCNEC, and regarding chemotherapy regimens, they showed a greater efficacy of platinum-etoposide chemotherapy in advanced stage with an ORR of 29%, including two cases of complete responses (CR) and four partial responses (PR); on the other hand, no cases of CR or PR were reported in patients treated with different chemotherapy schedules. Tokito et al. (83) compared the efficacy of SCLC-regimens in LCNEC patients and in so called ‘possible LCNEC’. The term ‘possible LCNEC’ was introduced by Travis et al. (84) referring to NSCLC with neuroendocrine IHC markers and neuroendocrine morphologic features on small samples derived from biopsies. They found no statistical differences in ORR, PFS, and OS between ‘pure LCNEC’ and ‘possible LCNEC’ groups. Considering the molecular subtypes of LCNEC (SCLC-like or NSCLC-like LCNEC) previously discussed, Rekhtman et al. demonstrated that most of patients with SCLC-like LCNEC responded to platinum-based regimens, while none of the patients with NSCLC-like LCNEC (25).

The greatest part of these studies does not show significant differences in the efficacy of SCLC-regimens in SCLC and LCNEC patients and, in clinical practice, a trend has been observed towards treating LCNEC patients in the same way as SCLC, rather than as NSCLC. Despite these results, Naidoo et al. assessed chemotherapy efficacy in 49 LCNEC patients, concluding that LCNEC may not respond to platinum/etoposide as strongly as cases of extensive stage SCLC (14). Accordingly, Zhuo et al. (43) confirmed that the response rate to platinum/etoposide in SCLC-like LCNEC patients is lower than historically reported for conventional SCLC.

Certainly, the small sample size of these studies reduces their results’ impact and underlies the heterogeneous biology of LCNEC, highlighting the emerging need of more extensive prospective clinical trials.

Some reports investigated the efficacy of other chemotherapic compounds; for example, pemetrexed efficacy in LCNEC was found to be very poor, may be due to higher levels of thymidylate synthase expressed by this histotype compared with other NSCLC subtypes (85, 86). In a prospective, multicenter, phase II trial chemotherapy-naïve advanced LCNEC, patients received everolimus in combination with paclitaxel and carboplatin for four cycles followed by maintenance everolimus until progression. The ORR was 45%, the median PFS 4.4 months, and the median OS 9.9 months. This study showed that everolimus in combination with chemotherapy could be an effective first-line treatment for patients with metastatic LCNEC (87). In another study, the authors categorized first-line chemotherapy for LCNEC patients into three groups: group I, which comprised gemcitabine, docetaxel, paclitaxel, or vinorelbine; group II, with pemetrexed treatment; and group III, which comprised etoposide chemotherapy. In patients with LCNEC, the group I chemotherapy resulted in a longer OS than group II and group III chemotherapy (median OS 8.5, 5.9, and 6.7 months respectively) (88). Nedaplatin is a newer platinum derivative that, co-administered with irinotecan, demonstrated promising effectiveness and safety in a retrospective analysis of 18 chemo-naïve patients with LCNEC (localized and advanced disease), but no prospective validation of this combination has been performed so far (89).

As for first-line treatment, also for second-line regimens there is not a standard of care for LCNEC patients. The most investigated drug in this setting is amrubicin, a fully synthetic topoisomerase II inhibitor, largely studied in SCLC patients (90). In a retrospective study, Yoshida et al. (91) reported the activity of amrubicin as single agent in second (72%) or subsequent lines of therapy (28%) in 18 LCNEC patients, showing ORR of 27.7%, median PFS 3.1 months, and OS 5.1 months.

Considering the reported efficacy of temozolomide (TMZ) in neuroendocrine tumors (92), a case report in literature describes the administration of TMZ in a woman with stage IV pretreated LCNEC, achieving PR after five cycles of TMZ treatment (93).

Galvano et al. have evaluated the prognostic and the predictive roles of systemic inflammatory biomarkers in 120 SCLC and LCNEC patients: neutrophil–lymphocyte ratio (NLR), lactate dehydrogenase (LDH), advanced lung cancer inflammation index (ALI), and the Lung Immune Prognostic Index (LIPI) score. At the multivariate analysis, Eastern Cooperative Oncology Group performance status, LDH levels and response after first-line chemotherapy were independently associated with OS. Median OS for good, intermediate, and poor LIPI was 15, 11, and 9 months, respectively (p = 0.091). Patients with higher NLR (>1.93) had an increased probability of tumor progression (p = 0.045). This study has provided evidence that levels of NLR, LDH, and ALI evaluated at diagnosis showed a significant prognostic role in lung neuroendocrine carcinomas (NEC), while LIPI stratified patients into three prognostic groups: good, intermediate and poor. Thus, systemic inflammatory biomarkers could facilitate the understanding of survival differences in the clinical management of lung NEC patients (94).

Unfortunately, the major conclusion from the above studies is that patients with advanced LCNEC have extremely poor prognosis and that the conflicting studies results do not allow a full agreement regarding the best treatment to use. Despite cytotoxic effects that many times are translated into tumor response, chemotherapy offers modest OS benefit and remains far from changing the natural history of advanced LCNEC (**Table 3**).

**Table 3:** Antineoplastic therapies in the first-line setting.

Authors	Study	Patients	Treatment	Outcomes
*Sun et al.*	Retrospective	45 LCNEC	SCLC-based (11) *vs* NSCLC-based (34)	mPFS 6.1 *vs* 4.9 m (p = 0.41) mOS 16.5 *vs* 9.2 m (p = 0.1)
*Le Treut et al.*	Prospective	42 LCNEC	Cisplatin–Etoposide	mPFS 5.2 m mOS 7.7 m
*Igawa et al.*	Retrospective	14 LCNEC, 77 SCLC	Platinum-based for SCLC; platinum and/or vinorelbine, docetaxel or irinotecan for LCNEC	mOS 10 m in LCNEC and 12.3 in SCLC
*Yamazaki et al.*	Retrospective	20 LCNEC	Cisplatin-based	Similar to SCLC
*Shimada et al.*	Retrospective	25 LCNEC vs 180 SCLC	Platinum-based CT/CRT	1y OS 34 *vs* 49% ORR 61 *vs* 63%
*Fujiwara et al.*	Retrospective	22 LCNEC	Platinum-based or paclitaxel	mOS 10.3 m
*Niho et al.*	Prospective	30 LCNEC, 10 SCLC, 1 NSCLC	Cisplatin–Irinotecan	RR 40% for LCNEC and 80% for SCLC mOS 12.6m for LCNEC and 17.3m for SCLC
*Rossi et al.*	Retrospective	83 LCNEC	Platinum–Etoposide *vs* other regimens	Best results with Platinum–Etoposide [ORR 29% (2 CR)]
*Tokito et al*	Retrospetive	10 “pure” LCNEC, 24 “possible” LCNEC	Platinum-based	No difference between possible and pure LCNEC
*Naidoo et al*	Retrospective	49 LCNEC	70% Platinum–Etoposide	ORR 37% for Platinum–Etoposide (worse than SCLC)
*Christopoulos et al*	Prospective	49 LCNEC	Carboplatin + Paclitaxel + Everolimus	ORR 45%, DCR 74% mPFS 4.4 m mOS 9.9 m
*Derks et al*	Retrospective	128 LCNEC	Gemcitabine, taxane or vinorelbine vs Pemetrexed *vs* platinum-etoposide	Gemcitabine, taxane or vinorelbine seemed to have better results
*Yoshiyuki et al*	Retrospective	18 LCNEC	Nedaplatin + Irinotecan	mOS 12.3 m

CRT, chemotherapy plus radiotherapy; RR, response rate; mOS, median overall survival; ORR, objective response rate; DCR, disease control rate; mPFS, median progression-free survival; CR, complete response.

## Potential Future Opportunities

### Immunotherapy

In the last decade, immunotherapy has dramatically changed the natural history of NSCLC improving OS and quality of life of these patients compared to standard chemotherapy (95). Recently, even for SCLC patients, immunotherapy (*i.e.*, atezolizumab) has been approved for first-line treatment thanks to results deriving from IMpower133 trial (96); durvalumab, in combination with platinum-based chemotherapy, has also been approved in the same setting of disease due to CASPIAN trial results (97). The main targets of checkpoint inhibitors include programmed death-receptor 1 (PD-1), its ligand (PD-L1) and Cytotoxic T-Lymphocyte Antigen 4 (CTLA-4). The PD-L1 is located on the tumor cell surface, and its interaction with the PD-1 receptor, expressed on activated T-cells, is known to suppress patient’s immune-response mechanisms to the tumor (98, 99). Due to the rarity of LCNEC, clinical trials evaluating immunotherapy efficacy have mainly included patients with adenocarcinoma or squamous cell carcinoma. In contrast, very poor information about PD-L1 expression and checkpoint inhibitors efficacy is available for LCNEC, and no clinical trials have been conducted for these patients. About 60% of pulmonary LCNEC do not exhibit the SCLC molecular signature (TP53 and RB1 co-mutation) which might explain the large percentage of LCNEC patients who are platinum-refractory or rapidly progress on a platinum regimen (25, 37). Although prospective data regarding use of immune checkpoint in LCNEC is lacking, small studies have evaluated PD-L1 expression and frequency in LCNEC patients, supporting further exploration of immune checkpoint in these patients (17).

Fan et al. (19) studied PD-1 and PD-L1 expression in pulmonary neuroendocrine tumors; of 10 patients with LCNEC, 100% were PD-L1 positive, and 80% were PD-1 positive. More recently, other studies analyzed PD-L1 expression, showing PD-L1 positivity in a minority of samples (20, 100). Kim et al. demonstrated that a substantial fraction of LCNEC (more than SCLC) has PD-L1 expression on tumor-infiltrating immune cells and that this expression is more frequently found in samples with higher mutation burden (101). The variability in percentages noted in these studies may be explained by the relatively small sample numbers of LCNEC cases employed. Nevertheless, comparing LCNEC with SCLC and low-grade neuroendocrine tumors, LCNEC exhibits a higher PD-L1 positivity, which is worth further investigations for potential immunotherapy application. Some studies were conducted to evaluate the correlation between PD-L1 expression and prognosis in patients with LCNEC. Eichhorn et al. (102) examined PD-L1 expression in 76 LCNEC patients, revealing that only 22% patients were positive for PD-L1 and, that positivity, was found on the tumor cell surface in 22% and within the tumor microenvironment (immune-cell infiltrate) in 36% of the patients. Furthermore, the authors demonstrated that PD-L1 expression has a prognostic role; indeed, poorer outcome was observed in patients with positive PD-L1 staining on the tumor cells and negative in the immune-cell infiltrate. On the contrary, negative tumors but a positive PD-L1 expression on immune-cell infiltrate was found to be connected with a better outcome (102). Hermans et al. investigated PD-L1 (scored positive if tumors showed ≥1% membranous staining) and CD8 expression (scored for intra-tumor T-cells and stromal cells) in advanced LCNEC, showing PD-L1 positivity in 16% of tumor samples. In contrast with the previous study, PD-L1 positivity and stromal/intra-tumor CD8 were correlated with superior OS (103). Similar results come from Tsuruoka et al. and Inamura et al. (20, 104) that showed better outcome in LCNEC patients with positive expression of PD-L1. In another multicenter retrospective study, respectively 11 and 75% of the tumor samples expressed PD-L1 on tumor (TCs) and immune cells (ICs), thus IC+TC− was the most frequent co-expression pattern. Median OS of metastatic LCNEC patients with the IC−TC+ profile was shorter than for those with the IC+TC− pattern, confirming Eichhorn results (105). Looking at these conflicting findings, it remains unclear whether PD-L1 could predict a good outcome in patients with LCNEC. Based on this background information, Ohtaki et al. performed a study to evaluate the relationship between outcome and expression of PD-L1, CD8, CD4, and Forkhead box protein P3 (Foxp3) in surgically resected LCNEC. They concluded that PD-L1 expression has a positive, but not statistically significant, impact on OS and recurrence free survival (RFS) of these patients, and that Foxp3 positive T-cells were an independent significant good prognostic factor for both OS and RFS; on the other hand, CD4 T-cells were an independent significant poor prognostic factor for RFS (106).

Since no clinical trials have ever been conducted for LCNEC patients, information related to immunotherapy efficacy only comes from small studies with a few patients treated with checkpoint inhibitors. Three cases of LCNEC managed at the University of Kentucky with immunotherapy were reported by Chauhan et al. (17); all of them were treated with nivolumab after platinum-based chemotherapy progression disease, achieving durable response with a complete radiological response or stable disease. Levra et al. presented their data about ten patients treated with checkpoint inhibitors (nine with nivolumab and one with pembrolizumab); 6/10 achieved partial response and 1/10 showed stable disease with a median PFS of 57 weeks (107). Wang et al. reported a single case of patient with stage IB LCNEC (PD-L1 negative but positive for PD-L1 amplification and tumor mutation burden high) who progressed after adjuvant chemotherapy after surgery; subsequently, the patient was treated with pembrolizumab, and after one cycle, all visible lesions shrunk, and no new lesions were seen. The patient remains on pembrolizumab with continued improvement of the disease 6 months after (108). Similarly, Zhang et al. described a case of a LCNEC patient who rapidly progressed after surgery and adjuvant chemotherapy but achieved complete response during nivolumab treatment probably due to high tumor mutational burden (TMB), although PD-L1 was negative (109). Daido et al. presented two cases of LCNEC treated with nivolumab as third and sixth lines of therapy for rapidly progressing disease to the previous lines; also in this case, the patients reported a radiological response to checkpoint inhibitor therapy (110). Sato et al. (111) reported another case of stage IVB LCNEC without PD-L1 expression that responded to nivolumab as third-line treatment, maybe due to a high TMB previously reported as predictive of response to immunotherapy (108). Agar et al. reported the efficacy of nivolumab in 17 pretreated patients with stage III−IV LCNEC, showing a prolonged OS as second-line treatment or beyond (112).

Although the correlation with response to immune checkpoint inhibitors remains under investigation, all these studies about PD-L1 expression and immunotherapy efficacy in LCNEC are interesting, especially considering the scarcity of treatment options and potential therapeutic targets in this rare and aggressive malignancy. As already mentioned, clinical trials in rare tumors are difficult to conduct; thus available data are not enough to establish immunotherapy role in LCNEC. Prospective data regarding use of immune checkpoint inhibitors are strongly needed (**Table 4**).

**Table 4:** Immunotherapy data in LCNEC patients.

Authors	Type of Study	Number of LCNEC patients	Line of therapy	Treatment	Outcomes
*Wang et al.*	Case report	1	1	Pembrolizumab	PR
*Zhang et al.*	Case report	1	1	Nivolumab	CR
*Chauhan et al.*	Case series	3	2	Nivolumab	DCR 100%
*Levra et al.*	Case series	10	2	Nivolumab (9/10) and Pembrolizumab (1/10)	PR 60% SD 10% mPFS 57 weeks
*Agar et al.*	Case series	17	≥2	Nivolumab	mOS 12.1 months ORR 29.4%
*Saito et al.*	Case report	1	3	Nivolumab	PR
*Daido et al.*	Case series	2	3−4	Nivolumab	PR

DCR, disease control rate; ORR, objective response rate; mPFS, median progression-free survival; CR, complete response; PR, partial response; SD, stable disease.

### Targeted Therapy

The identification of molecular aberrations leading to tumor growth and survival has dramatically changed the treatment landscape of NSCLC (113). Despite this complexity, cancer cell growth and survival can often be impaired by the inactivation of a single oncogene, so-called ‘driver mutation’. These mutations confer a growth advantage to the cells and have been positively selected during the cancer evolution. This phenomenon, called oncogene addiction, provides a rationale for molecular targeted therapy, that to date represents the gold standard treatment for ‘oncogene-addicted’ NSCLC (113). Unfortunately, driver mutations are extremely rare in ‘pure’ LCNEC while they occur more frequently in mixed forms of LCNEC-adenocarcinoma. Nevertheless, some cases of metastatic LCNEC carrying driver mutations (especially EGFR alterations) are reported, showing tyrosine kinase inhibitor (TKI) efficacy (114–116). The first report of LCNEC carrying an EGFR activating mutation and of gefitinib activity in LCNEC patients was published in July 2010; a 66-year-old woman who had never smoked was diagnosed with metastatic LCNEC. An exon 19 deletion of EGFR was detected, and a correlation between exon 19 deletion and LCNEC response to gefitinib was reported (21). Another case harboring an EGFR mutation showed a response to an EGFR-TKI in a LCNEC patient harboring an EGFR gene mutation responded for eight months to the EGFR-TKI icotinib (116). On the contrary, the histological transformation of EGFR-mutated NSCLC in LCNEC could represent a potential mechanism of resistance to TKI treatment (117).

Another driver alteration usually found in young and non-smoker NSCLC patients is anaplastic lymphoma kinase (ALK)-translocation (118), for which several TKIs have been developed and have dramatically changed prognosis of patients carrying this molecular rearrangement (119, 120). A few cases are described of LCNEC harboring ALK-translocation with conflicting TKI efficacy (23, 121).

With regard to other targets, anti-c-KIT, anti-VEGF, and anti-HER2 agents could be interesting new drugs for LCNEC treatment. Indeed, high expression rates of VEGF were found in LCNEC, supporting further assessment of anti-VEGF therapies in these patients. Likewise, strong expression of HER2 and c-KIT in a subset of patients suggests possible roles for targeted therapies, such as trastuzumab and imatinib, but clinical trials have never been performed and additional analyses are warranted (122).

As already mentioned, alterations in PI3K/AKT/mTOR pathway have been found in LCNEC, in particular PIK3CA mutations, PTEN loss, PIK3CA CNV, and RICTOR CNV (42, 44), but no compounds have been developed to target these potential driver alterations in LCNEC patients.

Also for DLL3, an inhibitory Notch-ligand highly expressed in SCLC and LCNEC, targeted therapy could represent a valid tool to improve LCNEC prognosis. Rovalpituzumab tesirine is a first-in-class antibody-drug conjugate directed against DLL3; its activity has been assessed in a phase I study including SCLC and LCNEC pretreated patients, showing an ORR of 18% and a manageable safety profile (123). In another study, Odate et al. (124) found that the expression of tropomyosin-related kinase B (TrkB) and brain derived neurotrophic factor (BDNF) was significantly higher in LCNEC than in SCLC, and they proposed that these two genes might be potential targets in LCNEC. Rossi et al. (72) conducted a study to achieve more accurate insight on the prognostic and possibly therapeutic value of the KIT receptors (RTKs), Platelet-derived growth factor receptor (PDGFR) *α*, PDGFR *β*, and mesenchymal epithelial transition factor (Met). Among these RTKs, only Met was significantly associated with patient survival at univariate analysis, but this data was not confirmed at multivariate analysis.

Based on these studies, prospective clinical studies on larger series of LCNEC are clearly mandatory to detect potential targetable molecular alterations. Further investigations are needed to develop targeted therapy for these patients.

## Conclusions

LCNEC is a rare and aggressive tumor. Due to its rarity and its likeness to other neuroendocrine tumors, histological diagnosis can be challenging (16, 30–32). Moreover, LCNEC management, especially in the advanced disease, is not clearly established. Physicians tend to treat patients with the same chemotherapy-regimens used for SCLC patients, achieving worse results in terms of ORR and OS (12–14). Nevertheless, according to LCNEC subtypes (SCLC-like LCNEC *versus* NSCLC-like LCNEC), chemotherapy-regimen choice should be driven by their molecular characteristics. Indeed, on the basis of the studies discussed, genomic subtyping represents a valid tool to predict prognosis and detect the best treatment for each LCNEC patient (43). With regard to potential future opportunities, some cases are reported of patients treated with checkpoint inhibitors, often achieving radiological and clinical response, in particular after platinum-based first-line treatment failure (107–111). A few data are also available concerning targeted therapy, due to a low frequency of driver mutations in LCNEC patients, but mostly of them have reported a moderate efficacy of TKIs in this subset of disease (114, 115, 119, 120).

To date, the therapeutic indications are mainly extrapolated from clinical practice, and no prospective clinical trials have been performed for LCNEC patients. Prognosis remains poor, even for early stages compared to NSCLC, regardless of which chemotherapy regimen is used. Therefore, a new paradigm in treating these patients is needed, and the inclusion of LCNEC in clinical trials is strongly recommended to identify the best therapeutic approach and to correlate biomolecular characteristics with the potential role of new treatment strategies.

## Author Contributions

MF, ASt, and EB conceived the original idea of the article, drafting and writing the paper. MS, SP, MMa, and FL revised the scientific content of specific sections of the manuscript and participated in drafting specific sections of the paper. EV, MC, AC, and ED’A participated in the critical revision of the paper. MS, SP, MMa, FL, RT, GR, ASc, SM, and MMi participated in the critical revision and editing of the manuscript. GT and EB conceived the original idea and provided critical revision of the manuscript as well as the final approval of the version to publish. All authors contributed to the article and approved the submitted version.

## Funding

Institutional funds of Università Cattolica del Sacro Cuore (UCSC).

## Conflict of Interest

The authors declare that the research was conducted in the absence of any commercial or financial relationships that could be construed as a potential conflict of interest.
